# The Addictive Model of Self-Harming (Non-suicidal and Suicidal) Behavior

**DOI:** 10.3389/fpsyt.2016.00008

**Published:** 2016-02-01

**Authors:** Hilario Blasco-Fontecilla, Roberto Fernández-Fernández, Laura Colino, Lourdes Fajardo, Rosa Perteguer-Barrio, Jose de Leon

**Affiliations:** ^1^Department of Psychiatry, Instituto de Investigación Sanitaria Puerta de Hierro (IDIPHIM), Puerta de Hierro University Hospital, Madrid, Spain; ^2^Centro de Investigación Biomédica en Red de Salud Mental (CIBERSAM), Madrid, Spain; ^3^Universidad Autónoma de Madrid, Madrid, Spain; ^4^Consulting Asistencial Sociosanitario (CAS), Madrid, Spain; ^5^Mental Health Research Center at Eastern State Hospital, Lexington, KY, USA

**Keywords:** suicidal behavior, non-suicidal self-injury, addiction, stress, opioid, dopamine

## Abstract

**Introduction:**

Behavioral addictions such as gambling, sun-tanning, shopping, Internet use, work, exercise, or even love and sex are frequent, and share many characteristics and common neurobiological and genetic underpinnings with substance addictions (i.e., tolerance, withdrawal, and relapse). Recent literature suggests that both non-suicidal self-injury (NSSI) and suicidal behavior (SB) can also be conceptualized as addictions. The major aim of this mini review is to review the literature and explore the neurobiological and psychological mechanisms underlying the addiction to self-harming behaviors.

**Method:**

This is a narrative review. The authors performed literature searches in PubMed and Google for suicidal behavior, self-harming, addiction, and “major repeaters.” Given the scarce literature on the topic, a subset of the most closely related studies was selected. The authors also focused on three empirical studies testing the hypothesis that major repeaters (individuals with ≥5 lifetime suicide attempts) represent a distinctive suicidal phenotype and are the individuals at risk of developing an addiction to SB.

**Results:**

The authors reviewed the concept of behavioral addictions and major repeaters, current empirical evidence testing concerning whether or not NSSI and SB can be understood as “addictions,” and the putative mechanisms underlying them.

**Conclusion:**

Our review suggests that both NSSI and SB can be conceptualized as addictions. This is relevant because if some individual’s self-harming behaviors are better conceptualized as an addiction, treatment approaches could be tailored to this addiction.

## Introduction

Behavioral addictions can be defined “as a process whereby a behavior […] is employed in a pattern characterized by loss of control and continuation despite significant negative consequences […]” (1). They are frequent, and share many characteristics and common neurobiological and genetic underpinnings with substance addictions (i.e., tolerance, withdrawal, and relapse) (2). Behavioral addictions include activities such as gambling, sun-tanning, shopping, Internet use, work, exercise, or even love and sex (3–10). Recent literature suggests that both repetitive non-suicidal self-injury (NSSI) and suicidal behavior (SB) could also be understood as “addictive behaviors” in some individuals (11–16). If confirmed, this might change the way we currently treat repetitive self-harming behaviors. For instance, in the same vein as alcoholics are treated with naltrexone, individuals characterized by repetitive self-harming behaviors could benefit from treatment regimens traditionally used for substance dependence. The present mini review is aimed at briefly examining the literature on this topic.

## Methods

Initially, the authors performed literature searches in PubMed for suicidal behavior, self-harming, addiction, and “major repeaters.” Given that there was not a single reference, we expanded our search to Google. We found just one reference by Ken Tullis. Later on, we also included PubMed and Google searches on self-harming and addiction. Given the scarce literature on the topic, a subset of the studies most closely related to our aim was selected. The authors also focused on three empirical studies testing the hypothesis published in 2012 by Blasco-Fontecilla (see later) that major repeaters (individuals with ≥5 lifetime suicide attempts) represent a distinctive suicidal phenotype and are the individuals at risk of developing an addiction to SB.

## Results

### Non-Suicidal Self-Injury as a Behavioral Addiction

There is substantive theoretical literature suggesting that NSSI can be understood as a behavioral addiction, but very few empirical studies testing this compelling hypothesis exist (16). For instance, Faye suggested that the emotional state preceding NSSI is similar to the aversive withdrawal symptoms experienced by drug users (17). Washburn et al. also reported that individuals displaying NSSI often have strong urges to self-injure (18). Furthermore, some authors have reported that endogenous opioids are reduced in individuals who engage in NSSI, but evidence contradicting this hypothesis is also found [see Victor et al. (16), for a review]. In 2002, Nixon et al. (15) explored whether NSSI could be explained under an addictive paradigm in adolescents displaying repetitive NSSI. They developed a self-report measure, adapted from the DSM-IV criteria for substance dependence, and reported that 81% endorsed more than five criteria. Victor et al. (16) stressed that, whereas both negative and positive reinforcement sustain substance use, “only negative reinforcement perpetuates NSSI.” These authors were of the opinion that the repetition of NSSI was better explained by emotional processes than by addiction mechanisms.

### Suicidal Behavior as a Behavioral Addiction

As for suicidal behavior, in 1998, Tullis proposed a theory of suicide addiction (19). He described individuals addicted to SB as having three characteristics: (1) childhood trauma, (2) mood disorders, and (3) multiple addictions. Until recently, the only study that empirically supported this hypothesis was a report of three cases (20). In 2012, we proposed that some suicide attempters (major repeaters, individuals with ≥5 lifetime suicide attempts) were indeed the individuals addicted to SB (14). The characterization of major repeaters has been a neglected area of research (12). In a seminal paper, Kreitman and Casey studied over 3,000 parasuicides. They arbitrarily divided individuals into “first-evers” (those with no previous parasuicide), “minor repeaters” (those with a lifetime history of 2–4 parasuicides), and “major or grand repeaters” (those with ≥5 lifetime parasuicides) (21). They also warned that the variables associated with “minor repetition” were not necessarily the same related to “major repetition” (21). Major repeaters represent around 10% of all suicide attempters (21–23). They are heavy consumers of health resources, pose a challenge to clinicians (21), and are at higher risk of suicide completion (24, 25). Later on, we tried to empirically test our hypothesis in a series of different samples (11–14).

In a first study, we aimed at better characterizing major repeaters and testing whether they represent a distinctive suicidal phenotype; compared with 335 non-major repeaters (<5 suicide attempts), major repeaters (*n* = 35) were more likely to be female, diagnosed with anorexia nervosa or substance dependence, and to have higher levels of trait anger with lower expression of anger (12).

In a second study, we demonstrated that, compared to non-major repeaters (*n* = 71), major repeaters (*n* = 11, 13%) more frequently endorsed automatic positive reinforcement (“To feel something, because you felt numb or empty”) as a way to explain their SB. In this study, all major repeaters and 93% of the remaining suicide attempters received at least one Axis I diagnosis, but there were no statistically significant differences between the groups. Borderline personality disorder was more frequently diagnosed among major repeaters. However, relieving emptiness (automatic positive reinforcement) was an important pathway, even more relevant than borderline personality disorder, to major repetition of suicide attempts in our study (11). This is important because, in contrast with the above-mentioned study suggesting that NSSI is perpetuated mainly through the removal of negative emotions (negative reinforcement) (16), our study suggests that major repetition of suicide attempts is perpetuated primarily through the generation of emotions (positive reinforcement; for instance, relieving emptiness, or raising the level of care or support from their relatives). Given that suicide attempts may replace self-mutilation, a type of NSSI, to regulate negative emotions in multiple-suicide attempters (26), it is possible that some major repeaters initially begin their “suicide career” with repetitive NSSI (negative reinforcement) and then replace it with repetitive SB (positive reinforcement).

In a third study, a cross-sectional study at Puerta de Hierro University Hospital (Madrid, Spain), we recruited 118 suicide attempters including 8 major repeaters (7%, all women) (13). We found that major repeaters “were more likely diagnosed with panic disorder without agoraphobia,” borderline personality disorder, and a history of psychiatric inpatient admission than non-major repeaters. We explored whether or not major repeaters are addicted to SB by using seven criteria modified from the DSM-IV criteria for substance dependence: (1) tolerance, (2) withdrawal, (3) loss of control, (4) problems in quitting/cutting down, (5) much time spent using, (6) substantial reduction in activities, and (7) adverse physiological/physical consequences (13). Total dependence on SB was diagnosed if the individual had three or more of the seven criteria in the last 12 months. In our study, 83% of major repeaters met criteria for total dependence on SB, which is pretty similar to the 81% of individuals displaying NSSI who endorsed more than five dependence criteria on NSSI (15). In this third study, we ran a backward stepwise logistic regression model to provide odds ratios between major repeater status and total dependence corrected by confounding variables (13). The model selected total dependence and age as the remaining significant variables in the last step. In other words, our study suggested that major repeaters were addicted to SB, and that our finding was probably not explained by the presence of borderline personality disorder (13).

**Figure 1:**
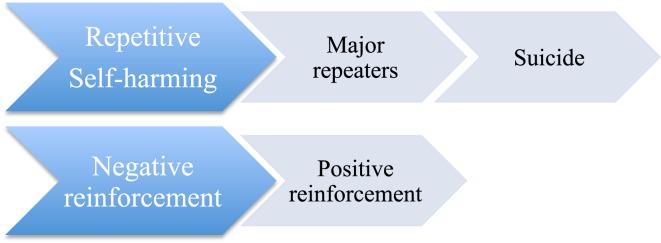
**Putative transition from repetitive NSSI to repetitive SB and the mechanisms involved**.

Here, it is important to refer to Stanley and colleagues; they have suggested that suicide attempters with a history of self-mutilation are a unique sub-population who use self-mutilation to deal with psychological pain (27). Unfortunately, their study involved DSM-IV, cluster B personality patients, and therefore, their findings could not be generalized. In any case, multiple-suicide attempters may use self-mutilation as a way of self-regulating negative emotions in the short term (26). However, in the long term, self-mutilating behaviors increase negative affectivity and become another stressor. Suicide attempts might then replace self-mutilation to regulate negative emotions in multiple-suicide attempters (26). In an adolescent study, suicide attempters, relative to suicide ideators, were less likely to display anger after a suicidal act (28). Figure 1 displays the putative transition from repetitive NSSI to repetitive SB and the mechanisms involved as suggested in the literature.

### Putative Mechanisms of the Addictive Hypothesis of Self-Harming Behaviors

**Figure 2:**
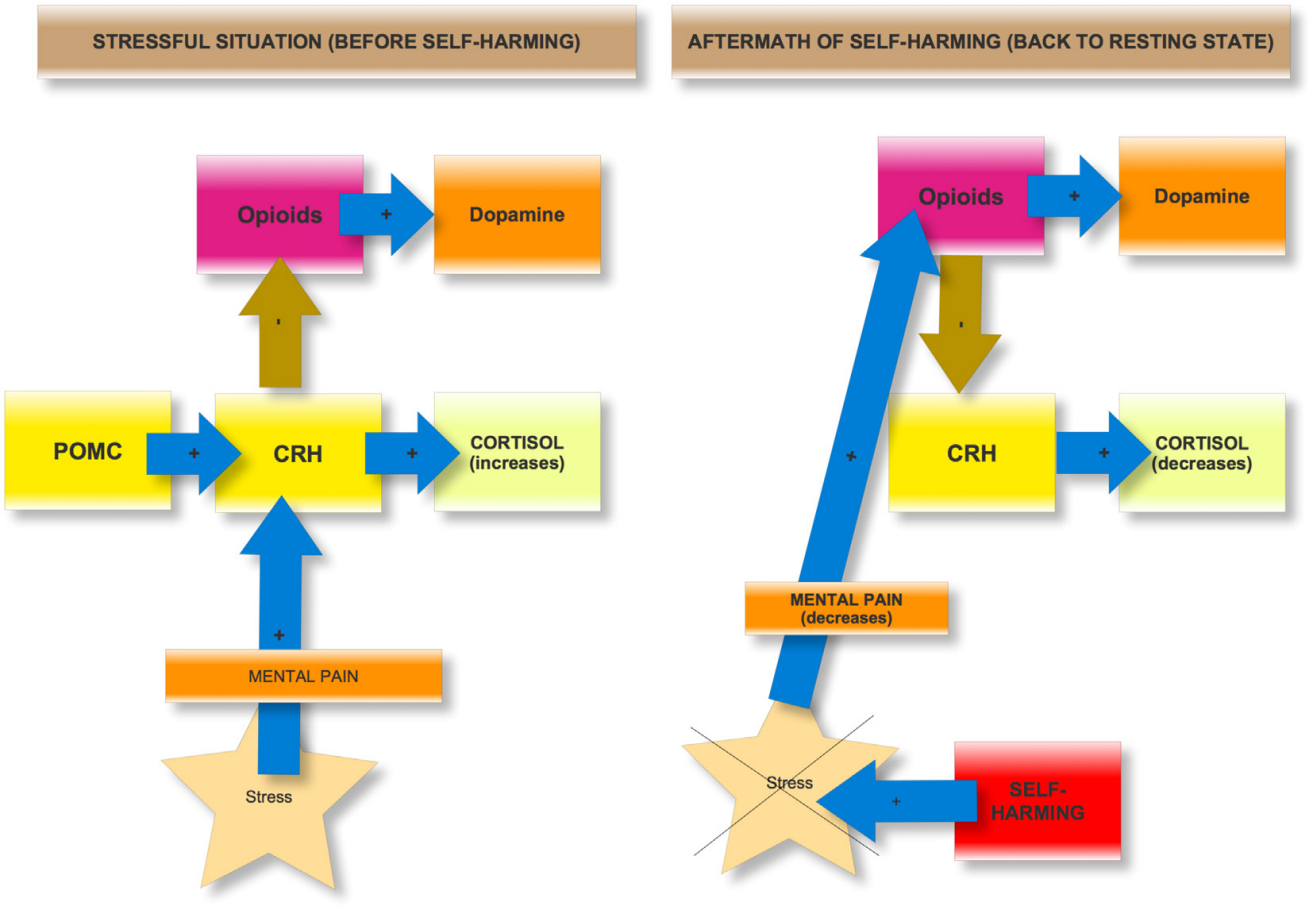
**Basic neurobiological mechanisms involved in the addiction to self-harming**.

The addiction to self-harming behaviors can be explained either by *neurobiological* or *psychological mechanisms*. Regarding *neurobiological mechanisms*, if self-harming behaviors can be addictive in some instances, it is reasonable to think that a compromised functioning of the brain’s motivational systems, including the mesocortical dopamine reward system and the endogenous opioid systems (29–31), and an overactivation of the stress system, are involved (29, 32). To the best of our knowledge, there are no studies directly relating the mesocortical reward system and self-harming behaviors. However, some authors have recently suggested that this system might be involved in the development of depression in a social defeat model of depression (30). Moreover, some authors demonstrated elevated endogenous opioid release following stressful events. For instance, chronic stress in mice produces opioid dependence (33), and prolonged mutilating elevates met-enkephalins (34). Given the role of psychological pain in suicide (35), and the growing evidence linking self-mutilation in particular and NSSI in general with the stress and opioid systems (27, 36), it is reasonable to think that the relief of psychological pain is probably associated with endogenous opioid release in the central nervous system in major repeaters. This opioid release may ultimately produce tolerance and addiction in vulnerable subjects (14). Furthermore, both acute and chronic stress increase the risk of using drugs (31), and corticotropin-releasing factor (CRF) is involved in the vulnerability to drug withdrawal (37) and relapse (38). Indeed, gene polymorphisms of the CRF receptors have been related to exacerbated stress responses and vulnerability to develop drug addiction (39). Furthermore, patients displaying repetitive NSSI were more likely to display lower levels of adrenocorticotropic hormone (ACTH) measured in the morning or evening (40). In other words, the hypothalamic–pituitary–adrenal (HPA) stress system (CRH, ACTH) and opioid systems (beta-endorphins) are closely related (41). This is not surprising, given that ACTH and β-endorphins are derived from the same precursor, pro-opiomelanocortin (POMC) (42, 43). In sum, opioid and dopaminergic systems, and HPA axis, which interact in the forebrain (31, 32) and can be activated either by psychoactive drugs or by behaviors (44), are probably involved in the development of an addiction to self-harming behaviors (Figure 2).

As for *psychological mechanisms*, Beck’s “sensitizing” hypothesis (45) and a cathartic effect (46) are probably involved in the addiction to self-harming behaviors. Beck (45) suggested that previous SB sensitizes suicidal thoughts and behaviors, such that they become more autonomous and easily precipitated. As self-harming episodes become more easily triggered by stressful life events, they also become more persistent and severe. Self-aggression ameliorates the emotional tension and painful emotions (i.e., emptiness) that precede SB (47–51). In a pilot fMRI study, the authors suggested that SB reduces mental pain (52). Beck’s “sensitizing” hypothesis of SB has gained some empirical support (53, 54). Furthermore, even after prolonged “free” periods, there is the risk of relapse, often precipitated by similar life events, in a way similar to that of drug addiction (55). The cathartic effect might be explained by either mobilization of interpersonal support (i.e., medical attention, caring family) (48, 51) or emotional venting of an unbearable emotional or physical state (48, 50). Indeed, self-harming behaviors can be used as a signaling strategy within the “bargaining model” of depression; self-harming behaviors would be a way to impose costs to the social group where there is a conflict (56).

### Therapeutic Implications

The addictive model of self-harming might have an important impact in the way we treat repetitive self-harming behaviors, and help in reducing the economic cost associated with them. The most evident targets for halting the development of an addiction to self-harming behaviors are the opioid and dopaminergic systems, and the HPA axis (37). In 1989, some advocated for clinical trials of opiate antagonists (i.e., naltrexone and buprenorphine) to treat NSSI (57). For instance, a recent yet unpublished controlled trial demonstrated that ultra-low-dose sublingual buprenorphine was effective in decreasing suicidal ideation (58). CRF receptor antagonists, particularly CRF1 antagonists (i.e., antalarmin), have also shown promising results for the treatment of drug abuse and addiction (39). CRF1 antagonists could have a lasting effect in blunting the elevated stress sensitivity in dependent individuals (39). As for the dopaminergic system, many animal models for self-injurious behavior share a compromised striatal dopamine system (59). Given that striatal dopamine receptors are coupled to L-type calcium channels, some have confirmed that calcium blockers, such as nifedipine, suppress self-injurious behavior in animal models (60).

Furthermore, any treatment alleviating psychological pain might also halt the development of the addiction to self-harming behaviors. For instance, lithium, known to have a specific “antisuicidal effect” (61), has an antinociceptive role probably mediated through the opioid system (62). In a still classic study, rats with brain lithium levels >0.5 mEq/L had a potentiation of endogenous enkephalin release (63). In another study, acute lithium enhanced “the morphine-induced analgesia in rats with or without chronic morphine treatment” (64). In any case, it is surprising that lithium’s putative antinociceptive action has been very poorly studied in the clinical arena. Moreover, medications acting on glutamatergic transmission, such as gabapentin, lamotrigine, topiramate, acamprosate, memantine, modafinil, d-cycloserine, and *N*-acetylcysteine, “are also of potential utility in the treatment of drug addiction, as well as various behavioral addictions such as pathological gambling” (65) and might also be useful in the treatment of addiction to self-harming behaviors.

Finally, new therapeutic pathways focused on treating either emptiness or psychological pain might also have a role in treating individuals addicted to self-harming behaviors. Regarding emptiness, some authors consider emptiness to be one of “the strongest precipitating factors in self-killing” (66). Quite surprisingly, as we have quoted before, there is little empirical research on the relationship between emptiness and SB (67). Some authors think that chronic feelings of emptiness are resistant to psychopharmacological agents (68). However, the cholinergic and serotonin systems might play a role in emptiness. For instance, some authors administered an acetylcholinesterase inhibitor – physostigmine – to individuals diagnosed with borderline personality disorder, other personality disorders, and healthy controls (69). Those individuals diagnosed with a personality disorder who displayed a depressive response to the physostigmine challenge were more likely to present a sense of emptiness. As we have previously suggested, drugs with anticholinergic activity, such as tricyclic or low potency antipsychotics, might prove useful in treating emptiness, among others (67). Regarding serotonin and emptiness, in 1998, Verkes and colleagues (70) discovered that patients meeting the criterion of “chronic feelings of emptiness and boredom” had the most elevated platelet serotonin in a sample of 144 consecutive recurrent suicide attempters. They suggested that some patients with borderline personality disorder, particularly when displaying elevated emptiness, might have a different pre-synaptic re-uptake of serotonin. Accordingly, their response to drugs acting *via* serotonin might be different.

As for psychological (mental) pain, more than 90% of suicide attempters report it (11). Indeed, we have recently proposed that what really unifies SB is psychological pain (71). Given the increasing evidence that physical and psychological pain share common neural pathways (72, 73), why shouldn’t we treat psychological pain using the same drugs that we use, for instance, for a headache? For the same reasons that we don’t use opioids for a headache (i.e., tolerance, abstinence, dependence), we shouldn’t use opioid agonists for individuals addicted to SB. However, non-steroidal anti-inflammatory drugs and acetaminophen could, *a priori*, be used. For instance, acetaminophen, taken during a 2-week period, reduced daily self-reported hurt feelings in comparison to a placebo (74, 75). Furthermore, there is increasing evidence that a single infusion of ketamine, an *N*-methyl-d-asparate antagonist, traditionally used as anesthetic, but also with analgesic properties, can lead to a rapid resolution of suicidal ideation in patients with treatment-resistant major depressive disorder (76). Moreover, we humans are a social species, and social peptides such as oxytocin, are involved in the development of different psychiatric disorders (77). Some self-harming behaviors are displayed in response to social problems and stressful life events, as previously stated. Given the critical role of oxytocin in bonding and stress, it is not surprising to find that low cerebrospinal fluid (CSF) oxytocin is related to high intent in suicide attempters (78). The use of an intranasal application of oxytocin might modify behavior. We might think that intranasal oxytocin could be used for some patients displaying self-harming behaviors in the aftermath of social problems; however, a recent editorial skeptically warned that “The wish to believe in the effectiveness of intranasal oxytocin appears to be widespread and needs to be guarded against with skepticism and rigor” (79). Finally, acceptance and commitment therapy has also proven effective in reducing psychological pain in suicide attempters (80), and therefore might be useful in halting the development of an addiction to self-harming behaviors.

## Conclusion

Our review suggests that both NSSI and SB can be conceptualized as addictions. This is relevant because if some individual’s self-harming behaviors are better conceptualized as an addiction, treatment approaches could be tailored to this addiction. The major limitation of the present review is that, given the space constraints, we could not expand our review to other interesting topics such as the putative role of impulsivity, a personality trait closely related to SB; impulsivity could be considered as the underpinning psychopathological substrate between mood, addiction, and self-injury behavior. Indeed, impulsivity has been proposed as the interface between mood and a number of addictive behaviors (81).

## Author Contributions

All authors: HB-F, RF-F, LC, LF, RP-B, and JL have made substantial contributions to the review, the drafting of the work, have approved the final version, and are accountable for all aspects of the work.

## Conflict of Interest Statement

In the last 3 years, Dr. Hilario Blasco-Fontecilla has received lecture fees from Eli Lilly, AB-Biotics, Janssen, and Shire. The remaining authors have no conflict of interest to declare.

## References

[1] Goodman A. Addiction: definition and implications. Br J Addict (1990) 85:1403–8. 10.1111/j.1360-0443.1990.tb01620.x 2285834

[2] Grant JE Brewer JA Potenza MN. The neurobiology of substance and behavioral addictions. CNS Spectr (2006) 11:924–30. 10.1017/S109285290001511X 17146406

[3] Goodman A. Sexual addiction: designation and treatment. J Sex Marital Ther (1992) 18:303–14. 10.1080/00926239208412855 1291701

[4] Cassin SE Von Ranson KM. Is binge eating experienced as an addiction? Appetite (2007) 49:687–90. 10.1016/j.appet.2007.06.012 17719677

[5] Favazza AR Suicide gestures and self-mutilation. Am J Psychiatry (1989) 146:408–9. 10.1176/ajp.146.3.408c 2919708

[6] Kourush AS Harrington CR Aadinoff B. Tanning as a behavioral addiction. Am J Drug Alcohol Abuse (2010) 36:284–90. 10.3109/00952990.2010.491883 20545604

[7] Reynaud M Karila L Blecha L Benyamina A. Is love passion an addictive disorder? Am J Drug Alcohol Abuse (2010) 36:261–7. 10.3109/00952990.2010.495183 20545601

[8] Sanchez-Carbonell X Beranuy M Castellana M Chamorro A Oberst U. [Internet and cell phone addiction: passing fad or disorder?]. Adicciones (2008) 20:149–59. 18551228

[9] Tantam D Whitakker J. Personality disorder and self-wounding. Br J Psychiatry (1992) 161:451–64. 10.1192/bjp.161.4.451 1393332

[10] Tao R Huang X Wang J Zhang H Zhang Y Li M. Proposed diagnostic criteria for Internet addiction. Addiction (2010) 105:556–64. 10.1111/j.1360-0443.2009.02828.x 20403001

[11] Blasco-Fontecilla H Baca-Garcia E Courtet P Garcia Nieto R De Leon J Horror vacui: emptiness might distinguish between major suicide repeaters and nonmajor suicide repeaters: a pilot study. Psychother Psychosom (2015) 84:117–9. 10.1159/000369937 25720355

[12] Blasco-Fontecilla H Jaussent I Olié E Béziat S Guillaume S Artieda-Urrutia P A cross-sectional study of major repeaters: a distinct phenotype of suicidal behavior. Prim Care Companion CNS Disord (2014) 16(4). 10.4088/PCC.14m01633 25664212 PMC4318672

[13] Blasco-Fontecilla H Artieda-Urrutia P Berenguer-Elias N Garcia-Vega JM Fernandez-Rodriguez M Rodriguez-Lomas C Are major repeater patients addicted to suicidal behavior? Adicciones (2014) 26:321–33. 25580865

[14] Blasco-Fontecilla H The addictive hypothesis of suicidal behavior. Med Hypotheses (2012) 78:350 10.1016/j.mehy.2011.11.005 22133558

[15] Nixon MK Cloutier PF Aggarwal S. Affect regulation and addictive aspects of repetitive self-injury in hospitalized adolescents. J Am Acad Child Adolesc Psychiatry (2002) 41:1333–41. 10.1097/00004583-200211000-00015 12410076

[16] Victor SE Glenn CR Klonsky ED Is non-suicidal self-injury an “addiction”? A comparison of craving in substance use and non-suicidal self-injury. Psychiatry Res (2012) 197:73–7. 10.1016/j.psychres.2011.12.011 22401975 PMC3625678

[17] Faye P. Addictive characteristics of the behavior of self-mutilation. J Psychosoc Nurs Ment Health Serv (1995) 33:36–9. 7666387 10.3928/0279-3695-19950601-08

[18] Washburn JJ Juzwin KR Styer DM Aldridge D. Measuring the urge to self-injure: preliminary data from a clinical sample. Psychiatry Res (2010) 178:540–4. 10.1016/j.psychres.2010.05.018 20580437

[19] Tullis K A theory of suicide addiction. Sex Addict Compulsivity (1998) 5:311–24. 10.1080/10720169808402339

[20] Mynatt S. Repeated suicide attempts. J Psychosoc Nurs Ment Health Serv (2000) 38:24–33. 11131404 10.3928/0279-3695-20001201-09

[21] Kreitman N Casey P. Repetition of parasuicide: an epidemiological and clinical study. Br J Psychiatry (1988) 153:792–800. 10.1192/bjp.153.6.792 3256378

[22] Barnes RA. The recurrent self-harm patient. Suicide Life Threat Behav (1986) 16:399–408. 10.1111/j.1943-278X.1986.tb00726.x 3798518

[23] Bille-Brahe U Kerkhof A De Leo D Schmidtke A Crepet P Lönnqvist J A repetition-prediction study on European parasuicide populations. Part II of the WHO/Euro Multicentre Study on Parasuicide in cooperation with the EC concerted action on attempted suicide. Crisis (1996) 17:22–31. 10.1027/0227-5910.17.1.22 8768403

[24] King MK Schmaling KB Cowley DS Dunner DL. Suicide attempt history in depressed patients with and without a history of panic attacks. Compr Psychiatry (1995) 36:25–30. 10.1016/0010-440X(95)90095-D 7705084

[25] Lewinsohn PM Rohde P Seeley JR. Psychosocial risk factors for future adolescent suicide attempts. J Consult Clin Psychol (1994) 62:297–305. 10.1037/0022-006X.62.2.297 8201067

[26] Esposito C Spirito A Boergers J Donaldson D. Affective, behavioral, and cognitive functioning in adolescents with multiple suicide attempts. Suicide Life Threat Behav (2003) 33:389–99. 10.1521/suli.33.4.389.25231 14695054

[27] Stanley B Sher L Wilson S Ekman R Huang YY Mann JJ. Non-suicidal self-injurious behavior, endogenous opioids and monoamine neurotransmitters. J Affect Disord (2010) 124:134–40. 10.1016/j.jad.2009.10.028 19942295 PMC2875354

[28] Negron R Piacentini J Graae F Davies M Shaffer D. Microanalysis of adolescent suicide attempters and ideators during the acute suicidal episode. J Am Acad Child Adolesc Psychiatry (1997) 36:1512–9. 10.1016/S0890-8567(09)66559-X 9394935

[29] Wise RA Koob GF. The development and maintenance of drug addiction. Neuropsychopharmacology (2014) 39:254–62. 10.1038/npp.2013.261 24121188 PMC3870778

[30] Nocjar C Zhang J Feng P Panksepp J. The social defeat animal model of depression shows diminished levels of orexin in mesocortical regions of the dopamine system, and of dynorphin and orexin in the hypothalamus. Neuroscience (2012) 30(218):138–53. 10.1016/j.neuroscience.2012.05.033 22626650

[31] Volkow ND Wise RA How can drug addiction help us understand obesity? Nat Neurosci (2005) 8:555–60. 10.1038/nn1452 15856062

[32] Lovallo WR. Cortisol secretion patterns in addiction and addiction risk. Int J Psychophysiol (2006) 59:195–202. 10.1016/j.ijpsycho.2005.10.007 16434116 PMC2257874

[33] Christie MJ Chesher GB. Physical dependence on physiologically released endogenous opiates. Life Sci (1982) 30:1173–7. 10.1016/0024-3205(82)90659-2 7201057

[34] Coid J Allolio B Rees LH. Raised plasma metenkephalin in patients who habitually mutilate themselves. Lancet (1983) 2:545–6. 10.1016/S0140-6736(83)90572-X 6136696

[35] Tossani E The concept of mental pain. Psychother Psychosom (2013) 82(2):67–73. 10.1159/000343003 23295405

[36] Hicks KM Hink SM. Concept analysis of self-mutilation. J Adv Nurs (2008) 64:408–13. 10.1111/j.1365-2648.2008.04822.x 19006819

[37] Kreek MJ Koob GF Drug dependence: stress and dysregulation of brain reward pathways. Drug Alcohol Depend (1998) 51:23–47. 10.1016/S0376-8716(98)00064-7 9716928

[38] Sarnyai Z Shamam Y Heinrichs SC. The role of corticotropin-releasing factor in drug addiction. Pharmacol Rev (2001) 53:209–43. 11356984

[39] Logrip ML Koob GF Zorrilla EP. Role of corticotropin-releasing factor in drug addiction: potential for pharmacological intervention. CNS Drugs (2011) 25:271–87. 10.2165/11587790-000000000-00000 21425881 PMC3273042

[40] Sandman CA Touchette PE Marion SD Chicz-Demet A. The role of proopiomelanocortin (POMC) in sequentially dependent self-injurious behavior. Dev Psychobiol (2008) 50:680–9. 10.1002/dev.20323 18688808 PMC2577125

[41] Traskman-Bendz L Ekman R Regnell G Ohman R HPA-related CSF neuropeptides in suicide attempters. Eur Neuropsychopharmacol (1992) 2:99–106. 10.1016/0924-977X(92)90018-4 1378770

[42] Dent RR Ghadirian AM Kusalic M Young SN Diurnal rhythms of plasma cortisol, beta-endorphin and prolactin, and cerebrospinal fluid amine metabolite levels before suicide. Case report. Neuropsychobiology (1986) 16:64–7. 10.1159/000118299 2438587

[43] Oquendo MA Sullivan GM Sudol K Baca-Garcia E Stanley BH Sublette ME Toward a biosignature for suicide. Am J Psychiatry (2014) 171:1259–77. 10.1176/appi.ajp.2014.14020194 25263730 PMC4356635

[44] Shaffer HJ Laplante DA Labrie RA Kidman RC Donato AN Stanton MV Toward a syndrome model of addiction: multiple expressions, common etiology. Harv Rev Psychiatry (2004) 12:367–74. 10.1080/10673220490905705 15764471

[45] Beck AT Beyond belief: a theory of modes, personality, and psychopathology. In: Salkovskis PM, editor. Frontiers of Cognitive Therapy. New York: Guildford Press (1996). p. 1–25.

[46] Farberow NL Personality patterns of suicidal mental hospital patients. Genet Psychol Monogr (1950) 42:3–79. 14778509

[47] Davis AT. Short-term course of depression following attempted suicide: a preliminary report. Acta Psychiatr Scand (1990) 81:345–51. 10.1111/j.1600-0447.1990.tb05462.x 2343762

[48] Jallade C Sarfati Y Hardy-Bayle MC. Clinical evolution after self-induced or accidental traumatism: a controlled study of the extent and the specificity of suicidal catharsis. J Affect Disord (2005) 85:283–92. 10.1016/j.jad.2004.11.002 15780698

[49] Sarfati Y Bouchaud B Hardy-Bayle MC. Cathartic effect of suicide attempts not limited to depression: a short-term prospective study after deliberate self-poisoning. Crisis (2003) 24:73–8. 10.1027//0227-5910.24.2.73 12880225

[50] Van praag H Plutchik R. An empirical study on the “cathartic effect” of attempted suicide. Psychiatry Res (1985) 16:123–30. 10.1016/0165-1781(85)90005-8 3865252

[51] Walker RL Joiner TE Jr Rudd MD. The course of post-crisis suicidal symptoms: how and for whom is suicide “cathartic”? Suicide Life Threat Behav (2001) 31:144–52. 10.1521/suli.31.2.144.21514 11459247

[52] Reisch T Seifritz E Esposito F Wiest R Valach L Michel K. An fMRI study on mental pain and suicidal behavior. J Affect Disord (2010) 126:321–5. 10.1016/j.jad.2010.03.005 20434779

[53] Bradvik L Berglund M. Repetition of suicide attempts across episodes of severe depression. Behavioural sensitisation found in suicide group but not in controls. BMC Psychiatry (2011) 11:5. 10.1186/1471-244X-11-5 21214896 PMC3023739

[54] Joiner TE Jr Rudd MD. Intensity and duration of suicidal crises vary as a function of previous suicide attempts and negative life events. J Consult Clin Psychol (2000) 68:909–16. 10.1037/0022-006X.68.5.909 11068977

[55] Hyman SE. Addiction: a disease of learning and memory. Am J Psychiatry (2005) 162:1414–22. 10.1176/appi.ajp.162.8.1414 16055762

[56] Hagen EH The bargaining model of depression. In: Hammerstein P, editor. Genetic and Cultural Evolution of Cooperation. Cambridge, MA: MIT Press in Cooperation with Dahlem University Press (2003). p. 95–123.

[57] Konicki PE Sschulz SC. Rationale for clinical trials of opiate antagonists in treating patients with personality disorders and self-injurious behavior. Psychopharmacol Bull (1989) 25:556–63. 2698484

[58] Yovell Y Bar G Masiah M Baruch Y Briskman I Asherov J Ultra-low-dose buprenorphine as a time-limited treatment for severe suicidal ideation: a randomized controlled trial. Am J Psychiatry (2015). 10.1176/appi.ajp.2015.15040535 26684923

[59] Visser JE Bar PR Jinnah HA. Lesch-Nyhan disease and the basal ganglia. Brain Res Brain Res Rev (2000) 32:449–75. 10.1016/S0165-0173(99)00094-6 10760551

[60] Blake BL Muehlmann AM Egami K Breese GR Devine DP Jinnah HA. Nifedipine suppresses self-injurious behaviors in animals. Dev Neurosci (2007) 29:241–50. 10.1159/000096414 17047321 PMC2951318

[61] Ahrens B Muller-Oerlinghausen B. Does lithium exert an independent antisuicidal effect? Pharmacopsychiatry (2001) 34:132–6. 10.1055/s-2001-15878 11518473

[62] Banafshe HR Mesdaghinia A Arani MN Ramezani MH Heydari A Hamidi GA. Lithium attenuates pain-related behavior in a rat model of neuropathic pain: possible involvement of opioid system. Pharmacol Biochem Behav (2012) 100:425–30. 10.1016/j.pbb.2011.10.004 22009032

[63] Staunton DA Deyo SN Shoemaker WJ Ettenberg A Bloom FE. Effects of chronic lithium on enkephalin systems and pain responsiveness. Life Sci (1982) 31(16–17):1837–40. 10.1016/0024-3205(82)90223-5 7154836

[64] You ZD Li JH Song CY Lu CL He C. Oxytocin mediates the inhibitory action of acute lithium on the morphine dependence in rats. Neurosci Res (2001) 41(2):143–50. 10.1016/S0168-0102(01)00272-3 11591442

[65] Olive MF Cleva RM Kalivas PW Malcolm RJ. Glutamatergic medications for the treatment of drug and behavioral addictions. Pharmacol Biochem Behav (2012) 100:801–10. 10.1016/j.pbb.2011.04.015 21536062 PMC3154511

[66] Eskin M. The effects of religious versus secular education on suicide ideation and suicidal attitudes in adolescents in Turkey. Soc Psychiatry Psychiatr Epidemiol (2004) 39(7):536–42. 10.1007/s00127-004-0769-x 15243691

[67] Blasco-Fontecilla H de Leon-Martinez V Delgado-Gomez D Giner L Guillaume S Courtet P Emptiness and suicidal behavior: an exploratory review. Suicidol Online (2013) 4:21–32.

[68] Stoffers J Vollm BA Rucker G Timmer A Huband N Lieb K. Pharmacological interventions for borderline personality disorder. Cochrane Database Syst Rev (2010) 6:CD005653. 10.1002/14651858.CD005653.pub2 20556762 PMC4169794

[69] Steinberg BJ Trestman R Mitropoulou V Serby M Silverman J Coccaro E Depressive response to physostigmine challenge in borderline personality disorder patients. Neuropsychopharmacology (1997) 17(4):264–73. 10.1016/S0893-133X(97)00051-1 9326751

[70] Verkes RJ Van der Mast RC Kerkhof AJ Fekkes D Hengeveld MW Tuyl JP Platelet serotonin, monoamine oxidase activity, and [3H]paroxetine binding related to impulsive suicide attempts and borderline personality disorder. Biol Psychiatry (1998) 43(10):740–6. 10.1016/S0006-3223(97)00317-X 9606528

[71] de Leon J Baca-García E Blasco-Fontecilla H From the serotonin model of suicide to a mental pain model of suicide. Psychother Psychosom (2015) 84(6):323–9. 10.1159/000438510 26398763

[72] Meerwijk EL Ford JM Weiss SJ. Brain regions associated with psychological pain: implications for a neural network and its relationship to physical pain. Brain Imaging Behav (2013) 7:1–14. 10.1007/s11682-012-9179-y 22660945

[73] Ducasse D Courtet P OlieÅL E. Physical and social pains in borderline disorder and neuroanatomical correlates: a systematic review. Curr Psychiatry Rep (2014) 16:443. 10.1007/s11920-014-0443-2 24633938

[74] DeWall CN Hurt feelings? You could take a pain reliever. Harv Bus Rev (2011) 89(4):28–9. 21510517

[75] Dewall CN Macdonald G Webster GD Masten CL Baumeister RF Powell C Acetaminophen reduces social pain: behavioral and neural evidence. Psychol Sci (2010) 21(7):931–7. 10.1177/0956797610374741 20548058

[76] Diaz Granados N Ibrahim LA Brutsche NE Ameli R Henter ID Luckenbaugh DA Rapid resolution of suicidal ideation after a single infusion of an N-methyl-D-aspartate antagonist in patients with treatment-resistant major depressive disorder. J Clin Psychiatry (2010) 71(12):1605–11. 10.4088/JCP.09m05327blu 20673547 PMC3012738

[77] Meyer-Lindenberg A Tost H. Neural mechanisms of social risk for psychiatric disorders. Nat Neurosci (2012) 15(5):663–8. 10.1038/nn.3083 22504349

[78] Jokinen J Chatzittofis A Hellström C Nordström P Uvnäs-Moberg K Asberg M. Low CSF oxytocin reflects high intent in suicide attempters. Psychoneuroendocrinology (2012) 37(4):482–90. 10.1016/j.psyneuen.2011.07.016 21852050

[79] Leng G Ludwig M. Intranasal oxytocin: myths and delusions. Biol Psychiatry (2016) 79(3):243–50. 10.1016/j.biopsych.2015.05.003 26049207

[80] Ducasse D Rene E Beziat S Guillaume S Courtet P Olie E Acceptance and commitment therapy for management of suicidal patients: a pilot study. Psychother Psychosom (2014) 83:374–6. 10.1159/000365974 25323551

[81] Swann AC Dougherty DM Pazzaglia PJ Pham M Moeller FG. Impulsivity: a link between bipolar disorder and substance abuse. Bipolar Disord (2004) 6(3):204–12. 15117399 10.1111/j.1399-5618.2004.00110.x

